# Angiopoietin-like protein 3 blocks nuclear import of FAK and contributes to sorafenib response

**DOI:** 10.1038/s41416-018-0189-4

**Published:** 2018-07-23

**Authors:** Yi Bao, Fu Yang, Bing Liu, Tangliang Zhao, Zhipeng Xu, Ying Xiong, Shuhan Sun, Le Qu, Linhui Wang

**Affiliations:** 1 Department of Urology, Changzheng Hospital, Second Military Medical University, Shanghai, 200003 China; 2 Department of Medical Genetics, Second Military Medical University, Shanghai, 200433 China; 3 Department of Urology, Shandong Provincial Qianfoshan Hospital, Shandong University, Jinan, 250014 China; 4 Department of Urology, Jinling Hospital, Nanjing University Clinical School of Medicine, Nanjing, 210002 China

**Keywords:** Renal cell carcinoma, Diagnostic markers, Protein translocation

## Abstract

**Background:**

Poor drug response of sorafenib is a major challenge which reduces clinical benefit of renal cell carcinoma (RCC) patients. It is therefore of great clinical significance to elucidate the underlying mechanism to restore the therapeutic response to sorafenib.

**Methods:**

Angiopoietin-like protein 3 (ANGPTL3) protein levels were measured by western blot and immunohistochemistry in two cohorts of RCC patients. Loss-of-function and gain-of-function experiments were performed to investigate the biological roles of ANGPTL3 in response to sorafenib treatment in RCC cells. Human proteome microarray and immunoprecipitation analysis were performed to explore the molecular mechanisms underlying the functions of ANGPTL3.

**Results:**

ANGPTL3 was upregulated in sorafenib-responsive RCC, which correlated with clinically good sorafenib response. Knockdown of ANGPTL3 conferred sorafenib-tolerance traits to RCC cells, whereas overexpression of ANGPTL3 restored sorafenib sensitivity in RCC cells. Mechanistically, ANGPTL3 bound to Focal Adhesion Kinase(FAK) and restained sorafenib induced nuclear translocation of FAK, leading to attenuate the ubiquitination of p53, which contributed to cellular apoptosis and enhanced sorafenib response.

**Conclusions:**

ANGPTL3 may be a novel predictor for the response of sorafenib therapy in RCC patients, and a potential target in improving its therapeutic effect.

## Introduction

The incidence of renal cell carcinoma (RCC) is rising throughout the world. Approximately 20% of RCC patients present with advanced stage disease at the time of diagnosis, and in patients with localised RCC, nearly 30% will develop recurrence and metastasis after tumour resection.^1,2^ Recently, an improved understanding of RCC pathogenesis has led to the development of multiple kinase inhibitors, such as sorafenib, that have become the mainstay of therapeutic options for treating advanced RCC patients. Sorafenib has potent antitumour and anti-angiogenic activities due to its inhibition of the serine/threonine kinase Raf-1, receptor tyrosine kinase vascular endothelial growth factor receptor, platelet-derived growth factor receptor, FMS-like tyrosine kinase 3, Ret, and mast/stem-cell growth factor receptor (KIT).^3–5^ Although sorafenib has been shown to improve the prognosis of patients in large randomised phase III studies, 22% of patients failed to respond to early treatment with sorafenib because of intrinsic resistance, and most of the remaining patients develop drug resistance and tumour progression after 6–15 months of therapy.^6–9^ Several studies have proposed that the activation of escape pathways from RAF/MEK/ERK/STAT3^10–13^ possibly may result in sorafenib resistance, but the picture remains unclear. In addition, no effective treatment is available after drug resistance arises. On the other hand, few prognostic factors have been validated as predictive biomarkers of sorafenib response. Thus, it is urgent to elucidate the underlying mechanisms of sorafenib resistance and discover reliable biomarkers that can predict sorafenib response in RCC patients.

There is a family of proteins that are structurally similar to angiopoietins (ANGs). These proteins are known as angiopoietin-like proteins (ANGPTLs), which comprise eight proteins, ANGPTL1-8.^14^ However, ANGPTLs do not bind to the ANG receptor, Tunica interna endothelial cell kinase (Tie) 2, or to the related protein Tie1, which suggests that they may have different biological functions than ANGs. ANGPTLs participate in multiple biological processes, such as angiogenesis,^15,16^ haematopoietic stem cell expansion,^17^ inflammation^18^ and cancer progression.^19–22^ ANGPTLs, such as ANGPTL1^23^ and ANGPTL4,^24,25^ have also been reported as involved in targeting drug resistance. Nevertheless, the role of ANGPTLs in the regulation of sorafenib response in RCC remains unknown.

In this study, we validate that ANGPTL3 is highly expressed in sorafenib-responsive RCC tissues and could predict clinical benefits from sorafenib therapy. Moreover, ANGPTL3 is functionally required for sorafenib sensitivity of RCC. Further mechanistic study reveals that ANGPTL3 interacts with Focal Adhesion Kinase (FAK) to inhibit its nuclear translocation, which finally dampen the ubiquitination of p53. Overall, we discover that ANGPTL3 modulates sorafenib sensitivity in RCC via inhibiting FAK mediated p53 ubiquitination.

## Methods

### RCC patients and clinical samples

RCC patients who underwent surgical resections before adjuvant therapy in Changhai and Changzheng Hospital, Shanghai, China, from 2009 to 2016 were included in this study. Ten pairs of sorafenib-sensitive and non-sensitive RCC tissues were used for preliminary screening, and the detailed clinical characteristics of these patients are provided in Supplementary Table 1. The expression of ANGPTL3 mRNA in 68 cases of RCC was detected and analysed, along with sorafenib sensitivity. The detailed clinical characteristics of these patients are provided in Supplementary Table 2. To evaluate the correlation between ANGPTL3 level and sorafenib response, tumour tissues were collected from the biopsies or surgical specimens of 136 advanced clear cell RCC (ccRCC) patients between August 2006 and April 2016. These patients had received no systemic treatment before biopsy or radical nephrectomy. Patients in the sorafenib group (*n* = 70) received at least two cycles of sorafenib therapy, and patients in control group (*n* = 66) received no therapy. These tissues were constructed into a tissue microarray (Biochip Company Ltd, China), and ANGPTL3 level was determined by immunohistochemistry. The detailed clinical characteristics of these patients are listed in Supplementary Table 5 and Supplementary Table 6. The response to sorafenib of the RCC patients was determined by computed tomography (CT) or magnetic resonance imaging, clinical progression, or death, with the use of the Response Evaluation Criteria in Solid Tumors (RECIST).

### Cell lines and reagents

The human RCC cell lines (OS-RC-2, Caki-2, Caki-1, A498, 786-O, ACHN, 760-P, KETR-3) were obtained from the Chinese Academy of Sciences (Shanghai, China). A498 and ACHN cells were incubated in MEM(10-010-CV, Corning, United States) supplemented with 10% foetal bovine serum (FBS, 16000044, Gibco, United States) and other cells were incubated in RPMI-1640 (10-040-CV, Corning, United States) containing 10% FBS. Cells were grown as a monolayer on plastic cell culture dishes at 37 °C in a humidified atmosphere containing 5% CO_2_. Sorafenib and RITA (NSC 652287) were purchased from Selleck chemicals (China). MG132 and cycloheximide (CHX) was purchased from APExBIO (United States). The primers used were listed in Supplementary Table 8 and the antibodies used were listed in Supplementary Table 9.

### Animal studies

The animal studies were approved by the Institutional Animal Care and Use Committee of the Second Military Medical University, Shanghai, China. Male athymic BALB/c nude mice (4–5 weeks old) were used. A total of 3 × 10^6^ lv-ANGPTL3 and lv-NC OS-RC-2 cells was injected subcutaneously into left and right side of model respectively (*n* = 8). One week after the injection of tumour cells, animals were randomly assigned to the control or experimental groups (*n* = 4 mice/group). The mice were treated with either vegetable oil (control) or sorafenib (80 mg/kg in vegetable oil). Xenograft volumes were evaluated by caliper measurements of two perpendicular diameters and calculated individually as formula: Volume = a × b^2^/2 (a represent length and b represent width). Xenograft growth was measured weekly and quantified using a noninvasive bioluminescence In-Vivo Imaging System (IVIS; Xenogen) 10 min after intraperitoneal injection of 4.0 mg of luciferin (Gold Biotech) in 50 μl of saline, as previously described.^26^ Xenograft samples were collected for histologic evaluation (paraffin section) or snap-freezing in liquid nitrogen, and We collected blood samples from orbital sinus and the blood was fractionated by centrifugation, and serum was stored at −80 °C until ready for use.

### Plasmids construction

The full-length FAK mRNA sequence was obtained from the NCBI website (NM_153831.3). The different fragments of ANGPTL3 were designed as Fig. 4d. The fragment was obtained by Gene synthesis and cloned into the pcDNA3.1 vector (General Biosystems (Anhui) Co. Ltd.)

### Cell transfection

Transfection of plasmids was performed by using jetPEI (PolyPlus Transfection, France). Transfection of siRNA (100 nM, GenePharma, China) was performed by using Lipofectamine RNAiMAX (Invitrogen, USA). Sequences of siRNA and shRNA against specific targets were listed in Supplementary Table 10.

### Lentiviral packaging and transfection

Lentiviruses encoding human ANGPTL3 were constructed and produced by Obio Technology (Shanghai). OS-RC-2 and caki-2 cells were infected with lenti-ANGPTL3 or lenti-EGFP in a MOI of 100. 72 h later puromycin was added to get the stable transfected cell lines.

### Human proteome microarray assay

The HuProt microarray(CDI Laboratories, Inc.) was composed of 20,240 human full-length proteins with N-terminal glutathione S-transferase (GST) tags. The HuProt microarray assay was performed by Wayen biotechnologies (Shanghai), Inc. according to the following procedure. Human Proteome microarrays (HuProtTM 20 K) were blocked with blocking buffer (1% BSA in 0.1% Tween 20, TBST) for 1 h at room temperature with gentle agitation. ANGPTL3 protein (ab176028, abcam, USA) was labeled with biotin by the Antibody Array Assay Kit (Full moon Biosystems, Sunnyvale, CA), and then diluted to 0.01 mg/ml in blocking buffer and incubated on the blocked proteome microarray at room temperature for 1 h. The microarrays were washed three times for 5 min each time with TBST, incubated with streptavidin-Cy5 at 1:1000 dilution (Thermo Fisher Scientific, USA) for 1 h at room temperature and underwent three more 5-min washes. The microarrays were spun dry at 1500 rpm for 3 min and subjected to scanning with a Genepix 4000B (Axon Instruments, Sunnyvale, CA) in order for results to be visualised and recorded. A GenePix Pro 6.0 was used for data analysis.

### Co-Immunoprecipitation

Co-IP was performed as the manufacturer’s instructions (Pierce Co-Immunoprecipitation (Co-IP) Kit, Thermo Scientific). RCC cells with indicated treatment were used for one immunoprecipitation reaction. Briefly, cells were lysed in a series of buffers and centrifugation steps to obtain lysate supernatant. Indicated antibodies were covalently coupled onto an amine-reactive resin and used to bait the corresponding proteins.

### Nucleoprotein extraction

Subcellular fractionation was performed as the manufacturer’s instructions (Thermo Scientific). Briefly, cells were lysed in a series of buffers and centrifugation steps to obtain a non-nuclear fraction and an intact nuclear pellet, followed by further lysing to isolate nuclear proteins. Nuclear and non-nuclear fractions (40–100 µg) were separated by SDS–PAGE and transferred to nitrocellulose filter (NC) membranes

### Immunocytochemistry

RCC cells were plated in laser confocal special culture dishs at 30% confluence and treated with indicated reagents at indicated concentration for 48 h. Then, the cells were fixed with 4% paraformaldehyde solution for 15 min at room temperature, permeabilised with 0.4% Triton X-100 in PBS for 5 min, and then blocked with 1% BSA in PBS for 1 h at 37 °C. The blocked cells were incubated with anti-FLAG antibody (1:100, Sigma) and anti-FAK antibody (1:100, abcam) overnight at 4 °C, followed by incubation with Alexa Fluor 488-conjugated anti-mouse IgG antibody

and Alexa Fluor 555-conjugated anti-rabbit IgG antibody (1:100, Invitrogen, Carlsbad, CA) for 2 h. Nuclear staining of cells was conducted using 4,6-diamidino-2-phenylindole (DAPI). Representative images were acquired using the Leica Microsystem.

### Immunohistochemistry

Specimens were stained with antibody ANGPTL3 (abcam, 1:100). The sections were heated at 70 °C for 1 h, dewaxed in xylene, and dehydrated through a gradient concentration of alcohol. After retrieving and blocking the endogenous peroxidase and nonspecific staining with 3% H_2_O_2_ and normal bovine serum, the sections were incubated with primary antibody overnight at 4 °C. The slides were then incubated with horseradish peroxidase (HRP)-conjugated secondary antibody for 10 min at 37 °C. Finally, the sections were visualised by diaminobenzidine (DAB) solution and counterstained with haematoxylin. Two pathologists blinded to the patient outcome scored the staining intensities and percentages of positive tumour cells independently.

### Data analysis

All statistical analyses in this study were performed with SPSS 22.0 software (SPSS Inc, USA). Data were presented as ‘mean ± sd’ The significance of mean values between two groups was analysed by two-tailed Student’s *t*-test. Spearman’s correlation analysis was performed to determine the correlation between two variables. Pearson chi-square test acted to analyse the clinical variables. Kaplan–Meier survival analysis was utilised to compare ccRCC patient survival based on dichotomised ANGPTL3 expression by log-rank test. Cox proportional hazards regression analyses were utilised to analyse the effect of clinical variables on patient survival. A *p* value of 0.05 was considered significant.

## Results

### ANGPTL3 is preferentially upregulated in sorafenib-responsive RCC

**Fig. 1:**
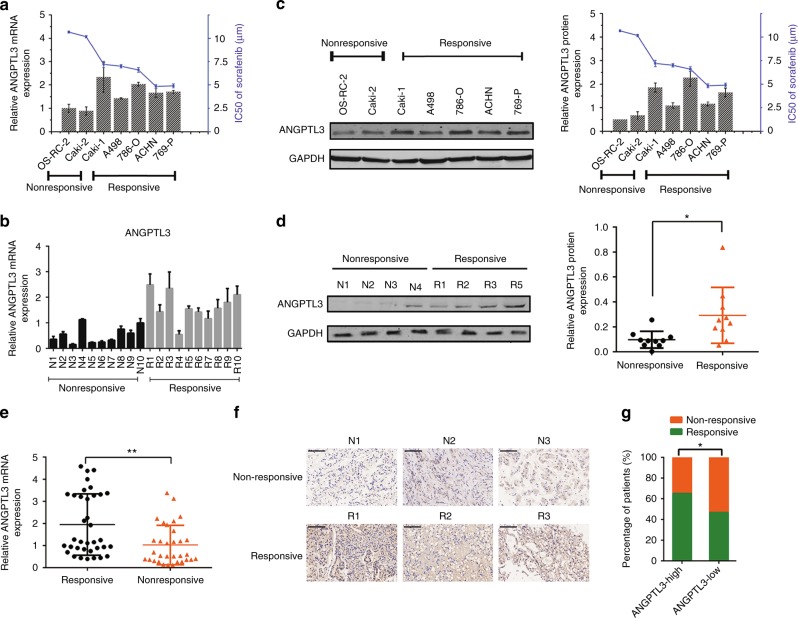
ANGPTL3 is preferentially upregulated in sorafenib-responsive renal cell carcinoma. **a** qRT-PCR analysis of ANGPTL3 mRNA levels and the IC50 of sorafenib in 7 RCC cell lines (*n* = 3). **b** The mRNA expression levels of ANGPTL 3 in an independent set of RCC tumours samples with 10 good and 10 poor responses to sorafenib therapy (*n* = 3). **c** Western blot analysis of ANGPTL3 protein level in the 7 RCC cell lines. On the right is a graph of ANGPTL3 relative expression and the IC50 of sorafenib (*n* = 3). **d** Representative western blot results for ANGPTL3 protein levels in the pretherapy tumour tissues from RCC patients with good responses (*n* = 10) or poor responses (*n* = 10) to sorafenib therapy. The chart on the right shows the relative expressions of ANGPTL3. **e** qRT-PCR analysis of ANGPTL3 mRNA levels in the pretherapy tumour tissues of RCC patients with good responses (*n* = 32) or poor responses (*n* = 36) to sorafenib therapy (p = 0.002). **f** Immunohistochemical analysis of ANGPTL3 protein level in RCC tissues before sorafenib therapy. Representative immunohistochemistry images from patients who were non-responsive (N1, N2, N3) and responsive (R1, R2, R3) to sorafenib are shown. The scale bar represents 100 μm. **g** Percentages of non-responsive and responsive to sorafenib samples between different ANGPTL3 levels. Results are presented as the means ± SD. *p < 0.05, **p < 0.01. See also Supplementary Figure 1 and Supplementary Table 1-2

To determine whether ANGPTLs are associated with sorafenib sensitivity in RCCs, we examined the mRNA expression levels of ANGPTL1-8 in 7 RCC cell lines (Fig. 1a and Supplementary Figure 1A), as well as a set of pre-treated tumour tissues from a cohort of RCC patients that presented with distinct responses to sorafenib therapy (Fig. 1b, Supplementary Figure 1B, Supplementary Table 1). Figure 1a, b shows that in both the RCC cell lines and the RCC tumour tissues, ANGPTL3 expression was correlated with the good response to sorafenib at the mRNA levels, which was further validatedat the protein levels by western blot (Fig. 1c, d). The sample size was then increased to verify the above findings.Patients with lower ANGPTL3 expression in RCC tissues had a higher proportion of sorafenib resistance (Fig. 1e–g, Supplementary Table 2). Together, these results showed that ANGPTL3 was preferentially upregulated in sorafenib-responsive RCC and may be required to maintain sorafenib sensitivity.

### ANGPTL3 is required to maintain sorafenib sensitivity in RCC cells

**Fig. 2:**
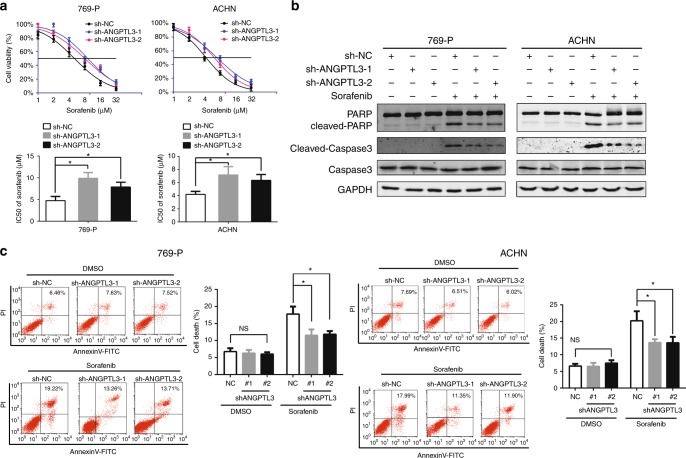
ANGPTL3 is required to maintain sorafenib sensitivity in RCC cells. **a** CCK8 assay of ACHN and 769-p cells transfected with sh-ANGPTL3-1, sh-ANGPTL3-2 or sh-NC after sorafenib treatment at the indicated concentrations for 48 h (*n* = 3). The IC50 values are shown in the lower histogram. **b** Western blot analysis of the indicated proteins in ACHN and 769-p cells transfected with sh-ANGPTL3-1, sh-ANGPTL3-2 or control sh-NC after sorafenib treatment at the indicated concentrations for 48 h (*n* = 3). GAPDH was used as a loading control. **c** Flow cytometry analysis of Annexin V-stained 769-p and ACHN cells transfected with sh-ANGPTL3-1, sh-ANGPTL3-2 or sh-NC after sorafenib treatment (5 μM) for 48 h (*n* = 3). Representative images (left) and average ratio of cell death (right) are shown. Results are presented as the means ± SD. *p < 0.05. See also Supplementary Figure 2

To explore the functional role of ANGPTL3 in sorafenib tolerance, we suppressed ANGPTL3 expression utilising two independent short hairpin RNAs against ANGPTL3 in two sorafenib responsive RCC cell lines (Supplementary Figure S2A and S2B). As shown in Fig. 2a, RCC cells with ANGPTL3 knockdown displayed elevated IC50 compared with the control cells. In addition, ANGPTL3 knockdown attenuated sorafenib-induced cellular apoptosis, as determined by levels of cleavage of poly (ADPribose) polymerase (PARP) and caspase 3 expression, as well as flow cytometry (Fig. 2b, c). Together, these data indicate that ANGPTL3 is required to maintain sorafenib sensitivity in RCC cells and inhibiting ANGPTL3 could increase tolerance to sorafenib treatment.

### Overexpression of ANGPTL3 enhances the sorafenib sensitivity of RCC in vitro and in vivo

**Fig. 3:**
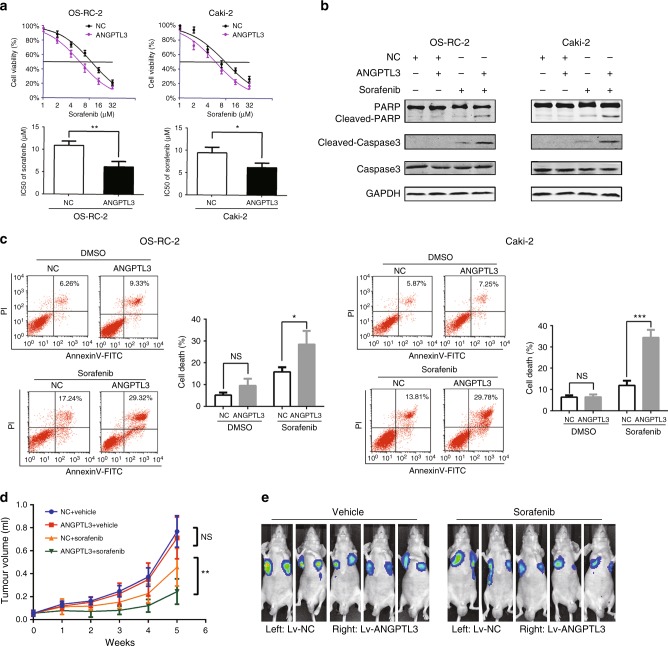
Overexpression of ANGPTL3 enhances the sorafenib sensitivity of RCC in vivo and in vitro. **a** CCK8 assay of ANGPTL3-overexpressing and control OS-RC-2 and Caki-2 cells after sorafenib treatment at the indicated concentrations for 48 h (*n* = 3). The IC50 values are shown in the lower histogram. **b** Western blot analysis of the indicated proteins in ANGPTL3-overexpressing and control OS-RC-2 and Caki-2 cells after sorafenib treatment at the indicated concentrations for 48 h (*n* = 3). GAPDH was used as a loading control. **c** Flow cytometry analysis of Annexin V-stained ANGPTL3-overexpressing and control OS-RC-2 and Caki-2 cells after sorafenib treatment (7.5 μM) for 48 h (*n* = 3). Representative images (left) and average ratios of cell death (right) are shown. **d** Nude mice were given xenografts of ANGPTL3-overexpressing and control OS-RC-2 cells (5 × 10^6^ cells per site) and were treated with vehicle or sorafenib (80 mg/kg). **e** Volumes of the tumours are shown (*n* = 5 per group). **e** Representative bioluminescent images. Results are presented as the means ± SD. *p < 0.05, **p < 0.01. See also Supplementary Figure 3

Next, we overexpressed ANGPTL3 expression in two sorafenib-resistant RCC cells (Supplementary Figure 3A and 3B). Compared with the control group, overexpressing ANGPTL3 resensitised RCC cells to sorafenib treatment and led to decreased IC50 (Fig. 3a). Increased cleavage of PARP and caspase 3 were observed in ANGPTL3 overexpressing RCC cells following sorafenib treatment (Fig. 3b). Consistently, flow cytometry showed that sorafenib exposure resulted in an increased proportion of apoptotic cells among ANGPTL3 overexpressing RCC cells (Fig. 3c).

Then we injected ANGPTL3-overexpressing and control OS-RC-2 cells subcutaneously into the left and right axils of nude mice, respectively. When the volume of the xenograft reached 100 mm^3^ mice were orally treated with vehicle or sorafenib (80 mg/kg/day). The results showed that xenografts formed from ANGPTL3-overexpressing RCC cells exhibited better responses to sorafenib (Fig. 3d, f and Supplementary Figure 3C). ANGPTL3 is a secreted protein, and we found that serum human ANGPTL3 levels was higher in tumour-bearing mice than in non-tumour-bearing mice (Supplementary Figure 3D), indicating that human ANGPTL3 was secreted from the xenograft. However, the different xenografts on the left and the right sides of the same mouse showed different response to sorafenib under the same blood ANGPTL3 concentration (Fig. 3e), suggesting that the ANGPTL3 in the bloodstream may not be the main factor influencing the response of tumour cells to sorafenib. Collectively, these findings indicate that the forced expression of ANGPTL3 overcomes sorafenib tolerance in RCC cells and that it may play its role through non-endocrine pathways.

### ANGPTL3 physically interacts with FAK

**Fig. 4:**
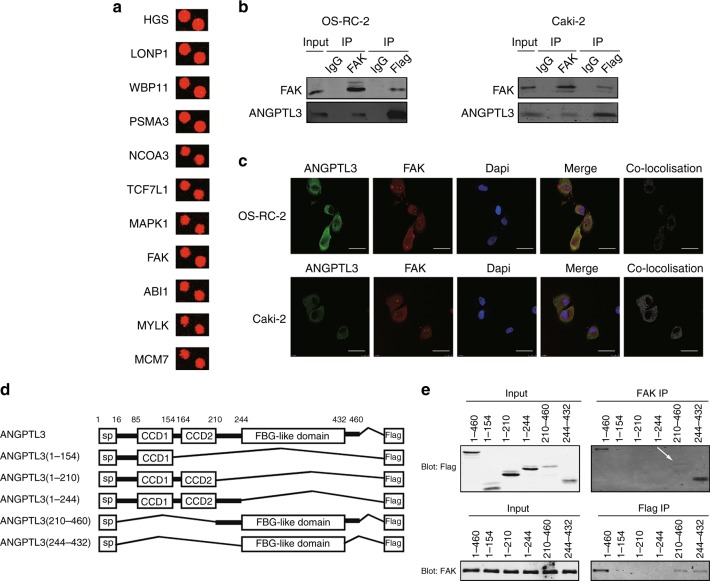
ANGPTL3 physically interacts with FAK. **a** Representative ANGPTL3-interacting proteins. Cy5 signals were measured at 635 nm. **b** Co-immunoprecipitation of ANGPTL3 and FAK in lysates of ANGPTL3-overexpressing OS-RC-2 and Caki-2 cells. **c** Immunofluorescence analysis of ANGPTL3 (green) and FAK (red) in OS-RC-2 and Caki-2 cells. The rightmost graphs show the co-localisation between the green signal (ANGPTL3) and the red signal (FAK). Pearson’s correlations were *R* = 0.8220 and 0.8262, respectively. Scale bar=25 μm. **d** Schematic diagram of different fragments of ANGPTL3. **e** Co-immunoprecipitation of Flag and FAK in lysates of OS-RC-2 cells transfected with plasmids containing different fragments of ANGPTL3. See also Supplementary Figure 4 and Supplementary Table 3 and 4

To dissect the mechanism underlying the promotive role of ANGPTL3 in the sorafenib sensitivity of RCCs, we used a human proteome microarray composed of 20,240 human full-length proteins with N-terminal glutathione S-transferase (GST) tags to seek ANGPTL3-interacting proteins (Supplementary Table 3) and there were 78 protein spots showing high signal (F635 > 500). The subcellular localisation of ANGPTL3 was measured and found to be mainly distributed in the cytoplasm (Supplementary Figure 4A-C). Cellular component analysis of Gene Ontology revealed that twenty-five of the above 78 proteins were located in the cytoplasm and 11 proteins were finally selected based on their functions for further evaluation (Fig. 4a, Supplementary Table 4). Then subjected to loss-of-function analysis (Supplementary Figure 4D). Four out of the 11 candidate proteins were associated with sorafenib sensitivity (Supplementary Figure 4E), but only one protein, FAK, was further reproducibly detected by independent immunoprecipitation (Fig. 4b and Supplementary Figure 4F). Consistently, ANGPTL3 co-localised with FAK in the cytoplasm by immunofluorescence staining and laser confocal observation (Fig. 4c); this result was validated by quantitative co-localisation analysis (Pearson’s correlations R = 0.8220 and 0.8262 respectively, in OS-RC-2 and Caki-2 cells).^27^ Then, we constructed truncated ANGPTL3 mutants to determine its binding sites with FAK (Fig. 4d). A co-immunoprecipitation assay revealed that the FBG-like domain of ANGPTL3 (residues 244-432) was required for its interaction with FAK (Fig. 4e). These data confirm that ANGPTL3 bind to FAK through its FBG-like domain.

### ANGPTL3 represses FAK-mediated sorafenib resistance

**Fig. 5:**
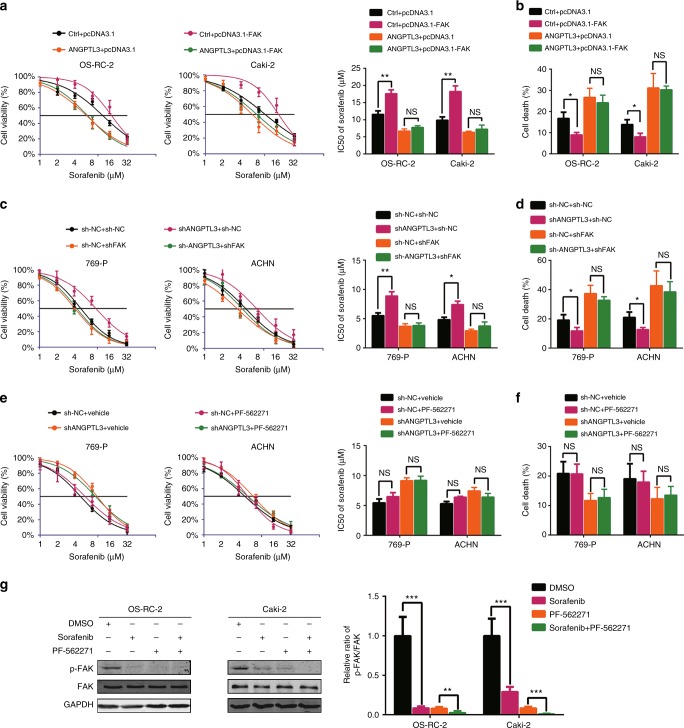
ANGPTL3 represses FAK-mediated sorafenib resistance. **a** CCK8 assay of ANGPTL3-overexpressing and control RCC cells transfected with the indicated plasmid after sorafenib treatment at the indicated concentrations for 48 h. The IC50 values are shown in the rightmost histogram. **b** Flow cytometry analysis of Annexin V-stained ANGPTL3-overexpressing and control RCC cells after sorafenib treatment for 48 h (*n* = 3). The average ratios of cell death are shown. **c** CCK8 assay of 769-p and ACHN cells transfected with sh-ANGPTL3-1, sh-FAK or sh-NC concurrent with sorafenib treatment at the indicated concentrations for 48 h (*n* = 3). The IC50 values are shown in the rightmost histogram. **d** Flow cytometry analysis of Annexin V-stained RCC cells transfected with shANGPTL3-1, sh-FAK or sh-NC for 48 h after sorafenib treatment for 48 h (*n* = 3). The average ratios of cell death are shown. **e** CCK8 assay of 769-p and ACHN cells transfected with sh-ANGPTL3-1 or sh-NC along with vehicle or PF-562271 (5 μM) concurrent with sorafenib treatment at the indicated concentrations for 48 h (*n* = 3). The IC50 values are shown in the rightmost histogram. **f** Flow cytometry analysis of Annexin V-stained RCC cells transfected with shANGPTL3-1 or sh-NC along with vehicle or PF-562271 (5 μM) for 48 h concurrent with sorafenib treatment (*n* = 3). The average ratios of cell death are shown. (**g**) Western blot analysis of the indicated proteins in OS-RC-2 and Caki-2 cells after sorafenib or PF-562271 treatment. The ratios of p-FAK/FAK are shown (*n* = 3) (right). Results are presented as the means ± SD. *p < 0.05, **p < 0.01, ***p < 0.001. See also Supplementary Figure 5

**Fig. 6:**
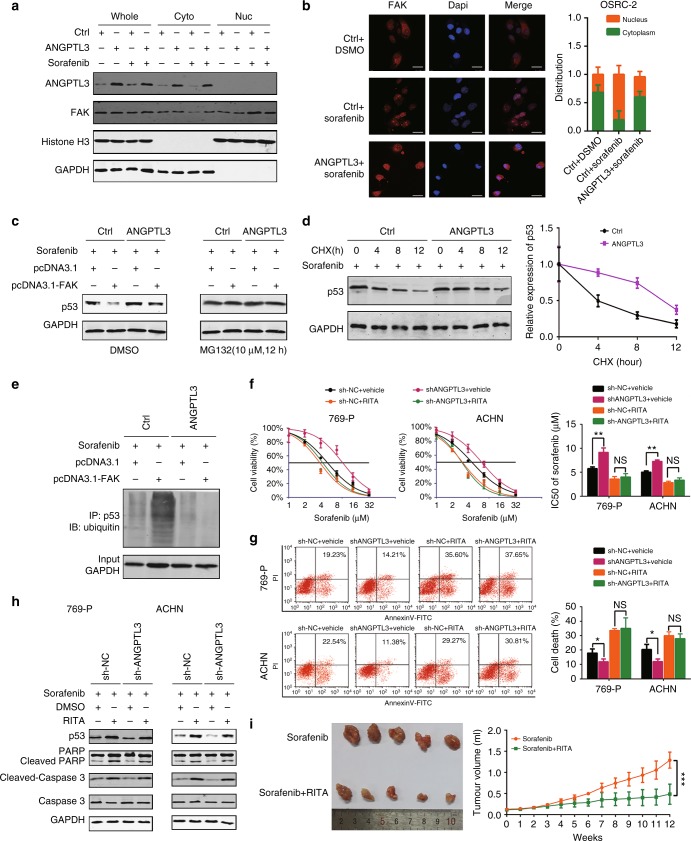
ANGPTL3 represses sorafenib resistance via inhibiting p53 ubiquitination. **a** Western blot analysis of FAK in the subcellular fractions of OS-RC-2 cells after sorafenib (7.5 μM) or DMSO treatment. **b** Immunofluorescence analysis of FAK (red) to determine its subcellular distribution in ANGPTL3-overexpressing or control OS-RC-2 cells after sorafenib (10 μM) treatment. Scale bar, 25 μm. FAK subcellular distribution was calculated (right). **c** Western blot analysis of protein p53 in ANGPTL3-overexpressing and control OS-RC-2 cells transfected with the indicated plasmid after sorafenib (7.5 μM) treatment for 48 h. The proteasome inhibitor MG132 was added 12 h prior to lysis. GAPDH was used as a loading control. **d** Western blot analysis of p53 in ANGPTL3-overexpressing and control OS-RC-2 cells after cycloheximide (CHX) and sorafenib treatment for various times. **e** Western blot analysis of protein p53 ubiquitination in ANGPTL3-overexpressing and control OS-RC-2 cells transfected with the indicated plasmid after sorafenib (7.5 μM) treatment for 48 h. GAPDH was used as a loading control. **f** CCK8 assay of 769-p and ACHN cells transfected with sh-ANGPTL3-1 or sh-NC along with vehicle or RITA concurrent with sorafenib treatment at the indicated concentrations for 48 h (*n* = 3). The IC50 values are shown in the rightmost histogram. **g** Flow cytometry analysis of Annexin V-stained RCC cells transfected with shANGPTL3-1 or sh-NC along with vehicle or RITA for 48 h concurrent with sorafenib treatment for 48 h (*n* = 3). Representative images (left) and average ratios of cell death (right) are shown. (H) Western blot analysis of the indicated proteins in 769-p and ACHN cells transfected with sh-ANGPTL3-1 or sh-NC along with DMSO or RITA concurrent with sorafenib (5 μM) treatment for 48 h. GAPDH was used as a loading control. **i** Nude mice were subcutaneously xenografted with OS-RC-2 cells (5 × 10^6^ cells) and treated orally with sorafenib (80 mg/kg) along with RITA (10 mg/kg) daily. Tumour volumes are shown (*n* = 5 per group). Results are presented as the means ± SD. *p < 0.05, **p < 0.01, ***p < 0.001. See also Supplementary Figure 6

FAK is a multifunctional regulator of cell signalling in various tumours and promotes cell motility, survival and proliferation.^28,29^ Overexpressing FAK reduced sorafenib effectiveness in RCC cells (Supplementary Figure 5A, B) and FAK knockdown improved the sorafenib sensitivity of RCC cells (Supplementary Figure 5C). Notably, overexpression of ANGPTL3 diminished the distinct difference in the sorafenib response between FAK-overexpressing and control RCC cells (Fig. 5a, b). Consistently, interfering with FAK also eliminated the discrepancy in sorafenib sensitivity between ANGPTL3-knocking down and control RCC cells (Fig. 5c, d). The most well-characterised mechanism that FAK promotes tumourigenicity involves FAK autophosphorylation at Y397. However we found that ANGPTL3 did not affect the total level and phosphorylation level of FAK (Supplementary Figure 5D). What’s more, PF-562271, an ATP-competitive kinase inhibitor of FAK, did not improve the sensitivity of RCC to sorafenib and did not reverse the resistance caused by ANGPTL3 knockdown (Fig. 5e, f). Sorafenib can inhibit FAK phosphorylation^30,31^ and we validated that in RCC cells (Fig. 5g). The simultaneous use of PF-562271 and sorafenib treatment increased the degree of FAK phosphorylation inhibition compared to PF-562271 or sorafenib treatment alone but the change of the degree is small. (Fig. 5g). In addition to its kinase-dependent functions, FAK functions through kinase-independent pathways, according to previous reports.^32,33^ Our results showed that in the absence of sorafenib, FAK was mainly cytoplasm localised, and after treatment with sorafenib, FAK appeared to have nuclear localisation, whereas overexpression of ANGPTL3 inhibited the nuclear localisation of FAK (Fig. 6a, b). Collectively, these data indicated that ANGPTL3 may repress FAK-mediated sorafenib resistance by inhibiting the nuclear localisation of FAK.

### ANGPTL3 inhibits FAK-mediated p53 ubiquitination

Nuclear FAK functions as a scaffold for p53 and MDM2, increasing p53 polyubiquitylation and degradation, thereby promoting cell survival.^34^ To verify that ANGPTL3 can modulate the kinase-independent function of FAK, we examined the expression and the ubiquitination of p53 after overexpressing ANGPTL3 or FAK. We found that overexpressing ANGPTL3 increased p53 protein level and reverse FAK-mediated p53 degradation while these differences disappeared after treatment with MG132, a proteasomal inhibitor (Fig. 6c). To further investigate whether ANGPTL3 could inhibit p53 protein degradation, a cycloheximide (CHX) chase experiment was performed, which demonstrated that overexpression of ANGPTL3 inhibited p53 protein degradation (Fig. 6d). The ubiquitination assay revealed that FAK enhanced sorafenib-induced ubiquitination of p53 and that overexpression of ANGPTL3 reversed this effect (Fig. 6e).These data demonstrated that ANGPTL3 inhibits FAK-mediated p53 ubiquitination via inhibiting sorafenib induced nuclear localisation of FAK. In addition, we overexpressed FAK and performed a CHX chase experiment with or without treatment of PF-562271 (Supplementary Figure 6A). Overexpression of FAK promoted the degradation of p53, whereas the combination of PF-562271 accelerated the degradation of p53, suggesting that FAK promotes ubiquitination of p53 independent of its kinase activity. It has been reported that FAK kinase inhibitors can promote the entry of FAK into nuclei,^35^ which was confirmed in the present study (Supplementary Figure 6B). These findings indicated that FAK kinase inhibitors such as PF-562271 could promote FAK nuclear entry similar to sorafenib, further explaining why PF-562271 and sorafenib did not produce a synergistic effect (Fig. 5E and F).

Then we used a p53 activator, RITA (NSC 652287), which inhibits the interaction between p53 and MDM2, thus inhibiting p53 ubiquitination. The results showed that RITA could promote sorafenib sensitivity in RCC cells and diminish the distinct difference in the IC50 of sorafenib and the cell death proportion after sorafenib treatment between ANGPTL3-knockdown and control RCC cells (Fig. 6f, g). RITA could increase the p53 protein level and restore the cleavage of PARP, cleavage of caspase 3 and p53 protein level in ANGPTL3-knockdown RCC cells (Fig. 6h). Moreover, combined RITA and sorafenib treatment prevented the emergence of sorafenib resistance, indicating a potential strategy for sorafenib resistance prevention (Fig. 6i). So ANGPTL3 or the interaction of p53 and MDM2 could serve as potential therapeutic targets to abrogate resistance to sorafenib therapy in RCC.

### High ANGPTL3 levels predict better responses to sorafenib in RCC patients

**Fig. 7:**
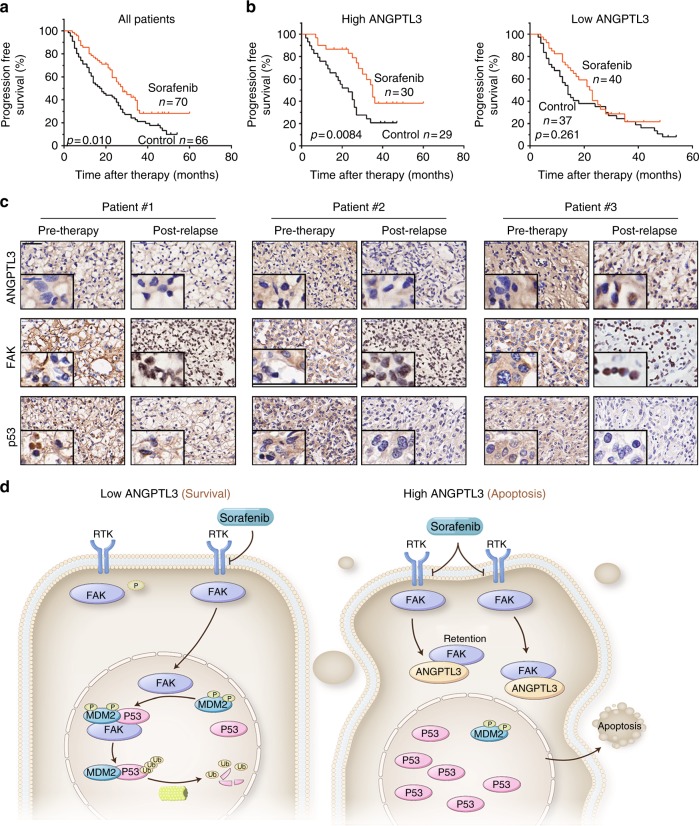
High ANGPTL3 levels predict better responses to sorafenib in RCC patients. **a**, **b** Kaplan–Meier analysis of PFS in RCC patients with and without sorafenib therapy. **a** All patients regardless of ANGPTL3 expression (*p* = 0.010). **b** patients with high levels (left, *p* = 0.0084) or low levels (right, *p* = 0.261) of ANGPTL3 expression. **c** Immunostaining of ANGPTL3, FAK and p53 in consecutive tumour sections from matched human RCC taken before sorafenib therapy (Pre-therapy) and after sorafenib resistance (Post-relapse). Black scale bar represents 50 μm. Micrograph indicates the magnified morphology of tumour tissue.Blue scale bar represents 10 μm. **d** Schematic diagram of the ANGPTL3-based signalling pathway in RCC sorafenib sensitivity. FAK enters the nucleus under sorafenib treatment and leads to ubiquitination of p53, thereby inhibiting cellular apoptosis; ANGPTL3 can reverse this process by binding to FAK and inhibiting its nuclear localisation. See also See also Supplementary Figure 7 and Supplementary Table 5-7

As ANGPTL3 is functionally involved in sorafenib response in RCC cells, we further evaluated whether the expression of ANGPTL3 in tumour tissues was associated with the response to sorafenib therapy. We measured ANGPTL3 levels in 136 RCC samples from 70 patients receiving sorafenib therapy and 66 patients receiving no drug therapy after surgery as control group (Supplementary Table 5). Sorafenib therapy provided limited benefits for the overall progression-free survival (PFS) of RCC patients (Fig. 7a), while patients with high ANGPTL3 expression levels in their tumours had a more significant improvement in PFS after receiving sorafenib than those in the control group did. However, patients with low ANGPTL3 expression levels showed poor response to sorafenib therapy (Fig. 7b). ANGPTL3 expression in this RCC cohort showed no differences in relation to different TNM stages or Furman grades (Supplementary Table 6). Both univariate and multivariate analyses revealed that sorafenib therapy was associated with significant improvements in PFS in patients with high ANGPTL3 expression levels (Supplementary Table 7).

Moreover, in clinically relaspsed tumour after sorafenib therapy, nuclear translocation of FAK and low expression of p53 were observed (Fig. 7c). In addition, the FAK and p53 protein levels also correlated with the IC50 value of sorafenib in the RCC cells (in Fig. 1a). In general, cells with higher FAK levels had higher IC50 values of sorafenib (except 786-O) and cells with lower p53 levels had higher IC50 values (except Caki-1 and 786-O) (Supplementary Figure 7A). Thus, the expression of ANGPTL3 could serve as an independent predictor of the response to sorafenib and the subcellular distribution of FAK, together with the expression of p53 might predict acquired resistance to sorafenib in RCC patients. In addition, the effects of several other TKI drugs were examined on FAK nuclear localisation. Sunitinib, pazopanib and cabozantinib had similar effects to those of sorafenib. All of these drugs promoted FAK nuclear entry, and overexpression of ANGPTL3 suppressed these effects (Supplementary Figure 7B), suggesting that the present findings may also apply to other TKI drugs.

## Discussion

An unmet clinical challenge exists in treating advanced RCC patients who are nonresponsive to sorafenib. The therapeutic options for these patients are quite limited at present. Hence, it is of great importance to investigate the mechanisms involved in sorafenib resistance and identify novel targets for sorafenib resistance prevention and therapy. In this study, we investigated the critical role of ANGPTL3 in sorafenib resistance and its underlying mechanism of action. We also demonstrated the value of ANGPTL3 as an independent predictor of response to sorafenib in RCC patients.

Many studies of the ANGPTLs family have been published, and these studies confirm that the ANGPTLs family is closely related to tumours. For example, ANGPTL1 can inhibit stemness, angiogenesis and metastasis in hepatocellular carcinoma cells,^36,37^ while ANGPTL2 and ANGPTL4 are upregulated in a variety of tumours and promote tumour proliferation and the process of epithelial-mesenchymal transition.^19,38^ Interestingly, in this study, by screening tumour tissues and RCC cell lines, we found that ANGPTL3 promoted sorafenib sensitivity in RCC, which is not consistent with the results of a few studies that suggest that ANGPTL3 promotes tumour development.^39,40^ ANGPTL3 is specifically expressed in the liver and kidney, and it has previously been reported that it binds to integrins to promote angiogenesis.^15^ It is generally believed that promoting angiogenesis can promote the development of tumours. However, in this study, the expression of ANGPTL3 and the functional experiments in vivo and in vitro indicate that ANGPTL3 promotes sorafenib sensitivity. This discrepancy is very interesting, and in vitro studies we found that ANGPTL3 did promote angiogenesis but that this effect could be eliminated under sorafenib treatment (data not shown). Therefore, we think that ANGPTL3 may play a role in other ways.

To find the downstream mechanisms of ANGPTL3 action, we used a human proteome microarray, which is more sensitive than other methods, to find that the protein FAK binds to ANGPTL3. Previous studies found that ANGPTL3 could combine with integrins,^15^ but the signal values of ANGPTL3–integrin binding in this microarray were low, which indicated that ANGPTL3 might not bind integrins. We hypothesise that the differences in the experimental environment between in vitro and in vivo and the difference in hybridisation times may have affected the outcome, indicating a limitation of the proteome microarray.

FAK is a non-receptor tyrosine kinase that transduces signals from a diverse group of stimuli (e.g., integrins, cytokines, chemokines, and growth factors) to control a variety of cellular pathways and processes, including cell proliferation, migration, morphology, and cell survival.^33,41–43^ It is generally believed to have both kinase-dependent and kinase-independent functions.^33^ This study found that sorafenib can promote the nuclear localisation of FAK and thus enhance the ubiquitination of p53, while ANGPTL3 can combine with FAK in the cytoplasm and inhibit nuclear localisation of FAK, thus activating p53 and promoting cellular apoptosis. Therefore, we believe that sorafenib has two effects on FAK: on the one hand, FAK phosphorylation was inhibited by sorafenib, reducing tumourigenicity^30,31^; the other hand, this study found that FAK entered the nuclear under sorafenib treatment, and led to the degradation of p53, promoting tumour survival. The nuclear function of FAK is a response by which RCC cells protect themselves against sorafenib, reducing the therapeutic efficacy of sorafenib. In addition, this study showed that sunitinib, pazopanib and cabozantinib can promote FAK nuclear entry, suggesting that this may be a common mechanism for RCC cells to respond to TKI drugs, which needs further study.

ANGPTL3 is a secreted protein, and previous studies have focused on its role in relation to target receptors on the cell membrane through the endocrine or paracrine pathway.^44,45^ However, we found that a considerable amount of the ANGPTL3 protein synthesised by RCC cells is still distributed inside these cells and can play a corresponding physiological role. This result adds to our understanding of ANGPTL3 function and suggests that the study of secreted proteins should not ignore their possible functions inside the cell.

The p53 gene is intact (i.e., not deleted, mutated, or methylated) in most RCC.^46^ P53 has been implicated as a master regulator of a variety of cellular processes, including proliferation, senescence, differentiation, apoptosis, ferroptosis, DNA repair, metabolism, angiogenesis, and autophagy.^47^ We found a decrease in p53 expression under sorafenib, consistent with previous reports.^48^ Designing small molecules to block the MDM2-p53 interaction and reactivate p53 function is a promising therapeutic strategy for the treatment of cancers retaining wild-type p53.^49^ In this study, we found that RITA, an activator of p53, can inhibit the occurrence of therapy resistance when combined with sorafenib. However, targeted drug resistance is associated with many factors, and as a secretory protein, ANGPTL3 does have the potential to act on stromal cells and thus affect the sensitivity of targeted therapy, a possibility that is not explored in this study and requires further study.

In conclusion, we showed that ANGPTL3 represses sorafenib resistance via regulating the ubiquitination of p53 by inhibiting the nuclear translocation of FAK (Fig. 7d). These data imply that ANGPTL3 may be not only a therapeutic target in the treatment of RCC patients with sorafenib resistance but also a novel biomarker for predicting responsiveness to sorafenib treatment.

## Electronic supplementary material

Supplementary Figure 1

Supplementary Figure 2

Supplementary Figure 3

Supplementary Figure 4

Supplementary Figure 5

Supplementary Figure 6

Supplementary Figure 7

Supplementary Figures legend clean vision

Supplementary table 1

Supplementary table 2

Supplementary table 3

Supplementary table 4

Supplementary table 5

Supplementary table 6

Supplementary table 7

Supplementary table 8

Supplementary table 9

Supplementary table 10

Supplementary Materials and Methods

## Availability of data and material

The datasets used and analyzed during the current study are available from the corresponding author on reasonable request.
