# Supplementary figures and images for: Angiopoietin-like protein 3 blocks nuclear import of FAK and contributes to sorafenib response
Source: Br J Cancer. 2018 Jul 23;119(4):450–61. doi: 10.1038/s41416-018-0189-4 (PMC6134083; doi:10.1038/s41416-018-0189-4)

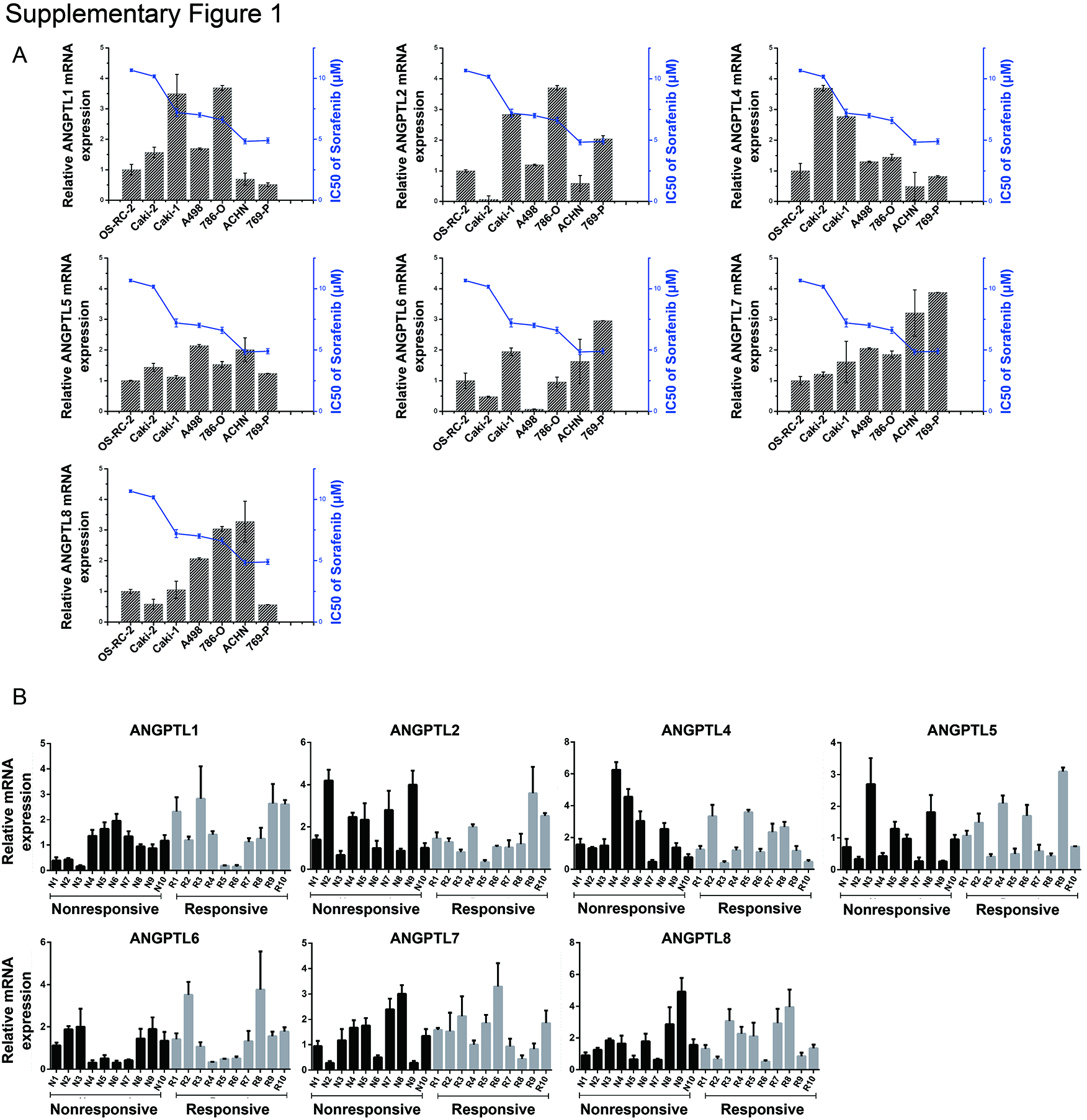

Supplement: Supplementary file 1 — Supplementary Figure 1 [file 41416_2018_189_MOESM1_ESM.tif]

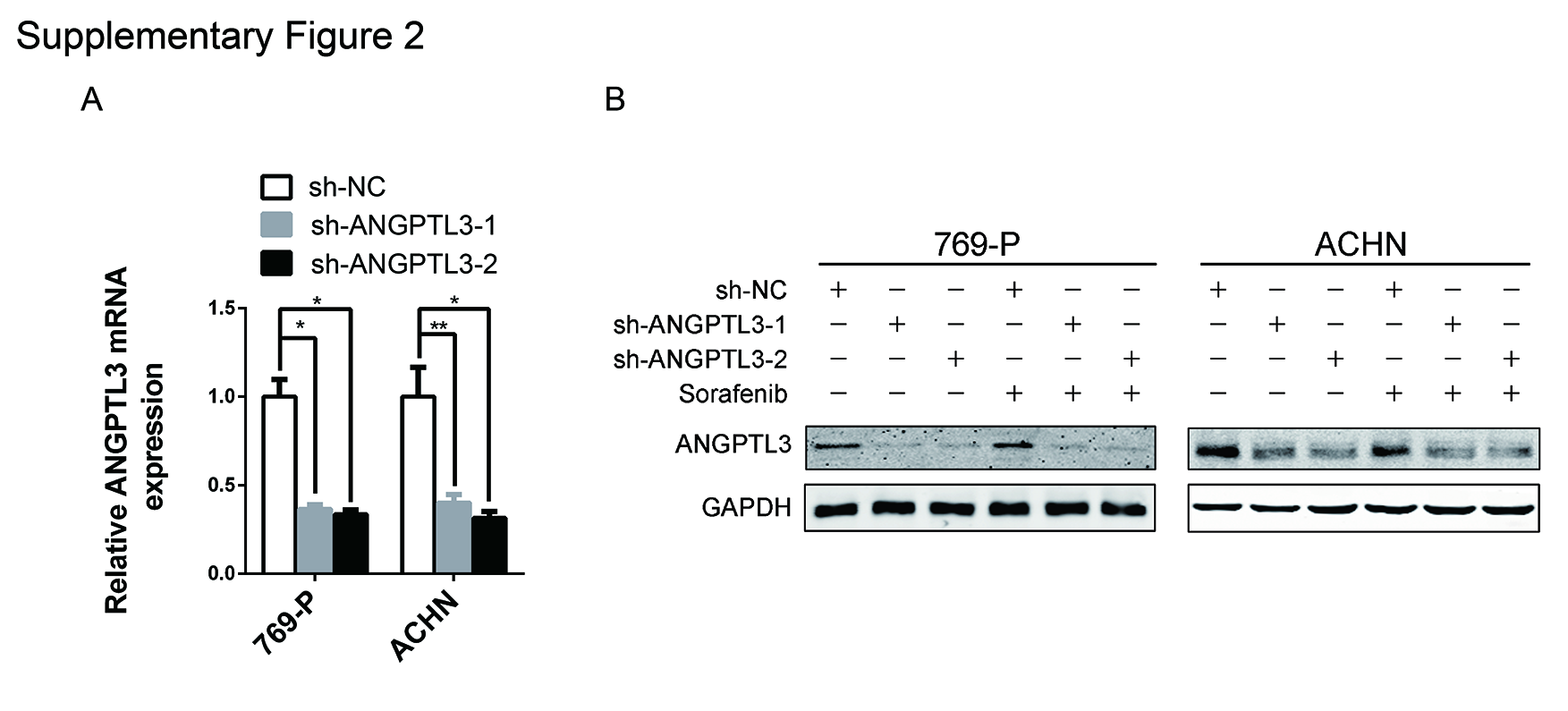

Supplement: Supplementary file 2 — Supplementary Figure 2 [file 41416_2018_189_MOESM2_ESM.tif]

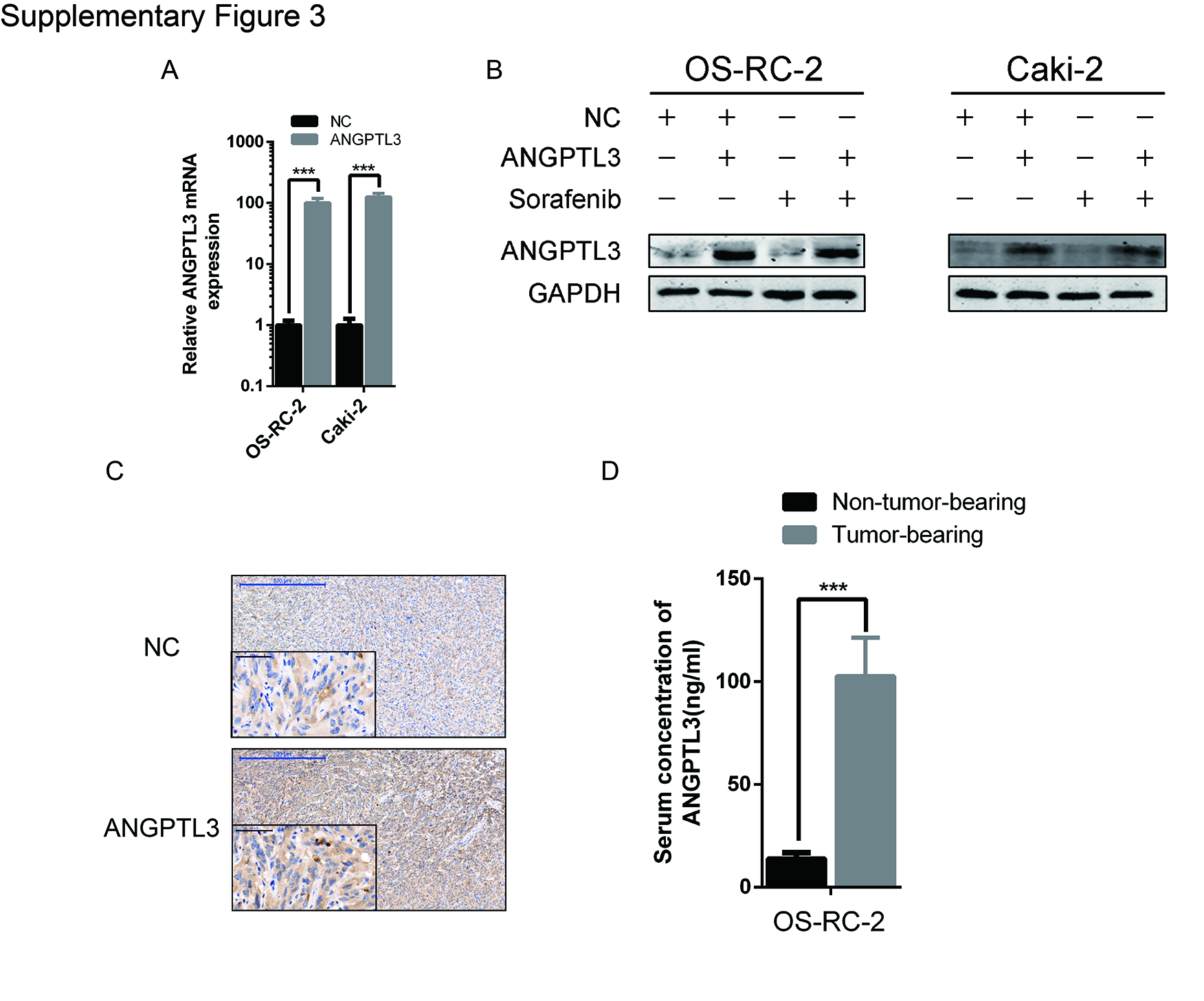

Supplement: Supplementary file 3 — Supplementary Figure 3 [file 41416_2018_189_MOESM3_ESM.tif]

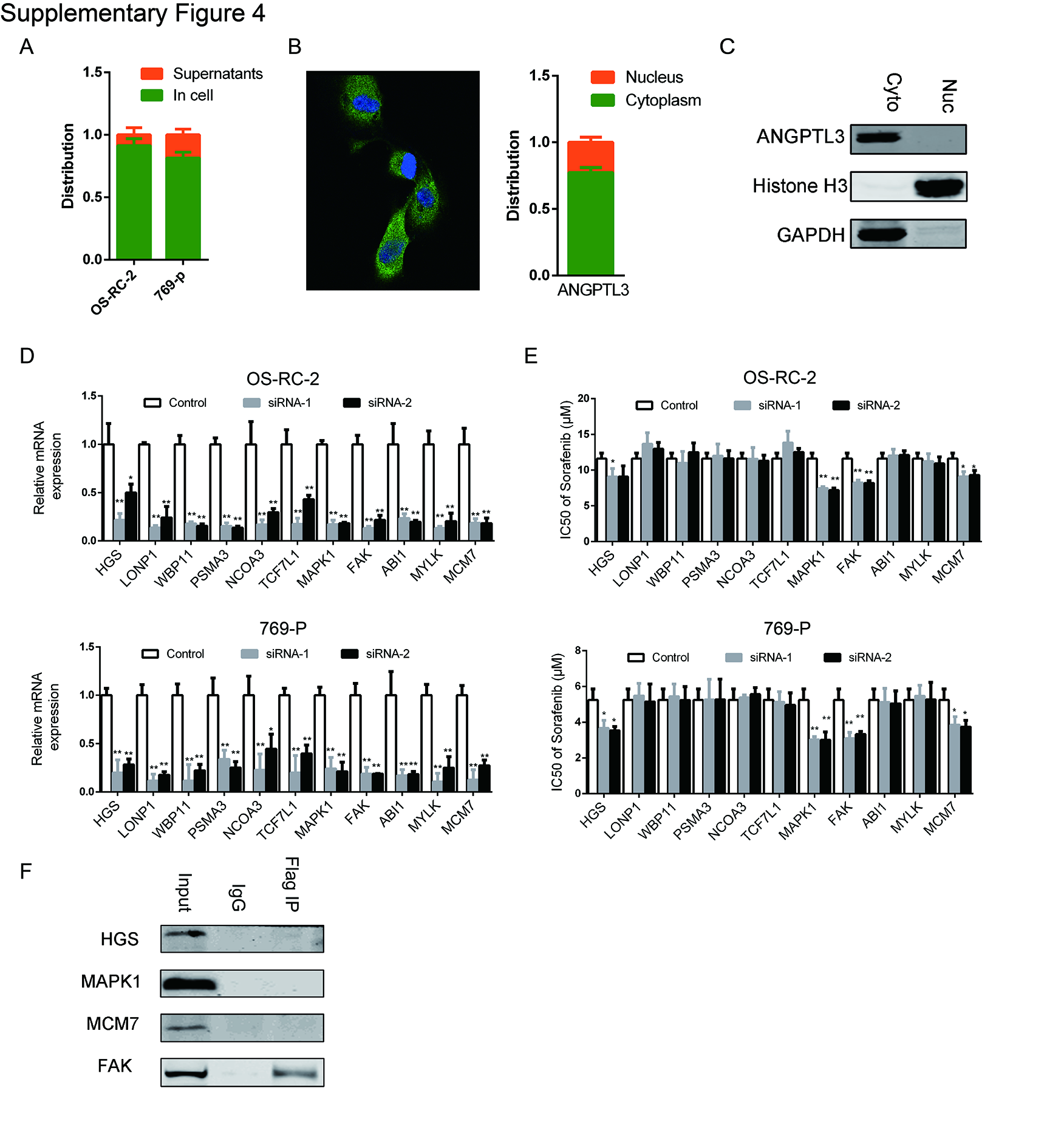

Supplement: Supplementary file 4 — Supplementary Figure 4 [file 41416_2018_189_MOESM4_ESM.tif]

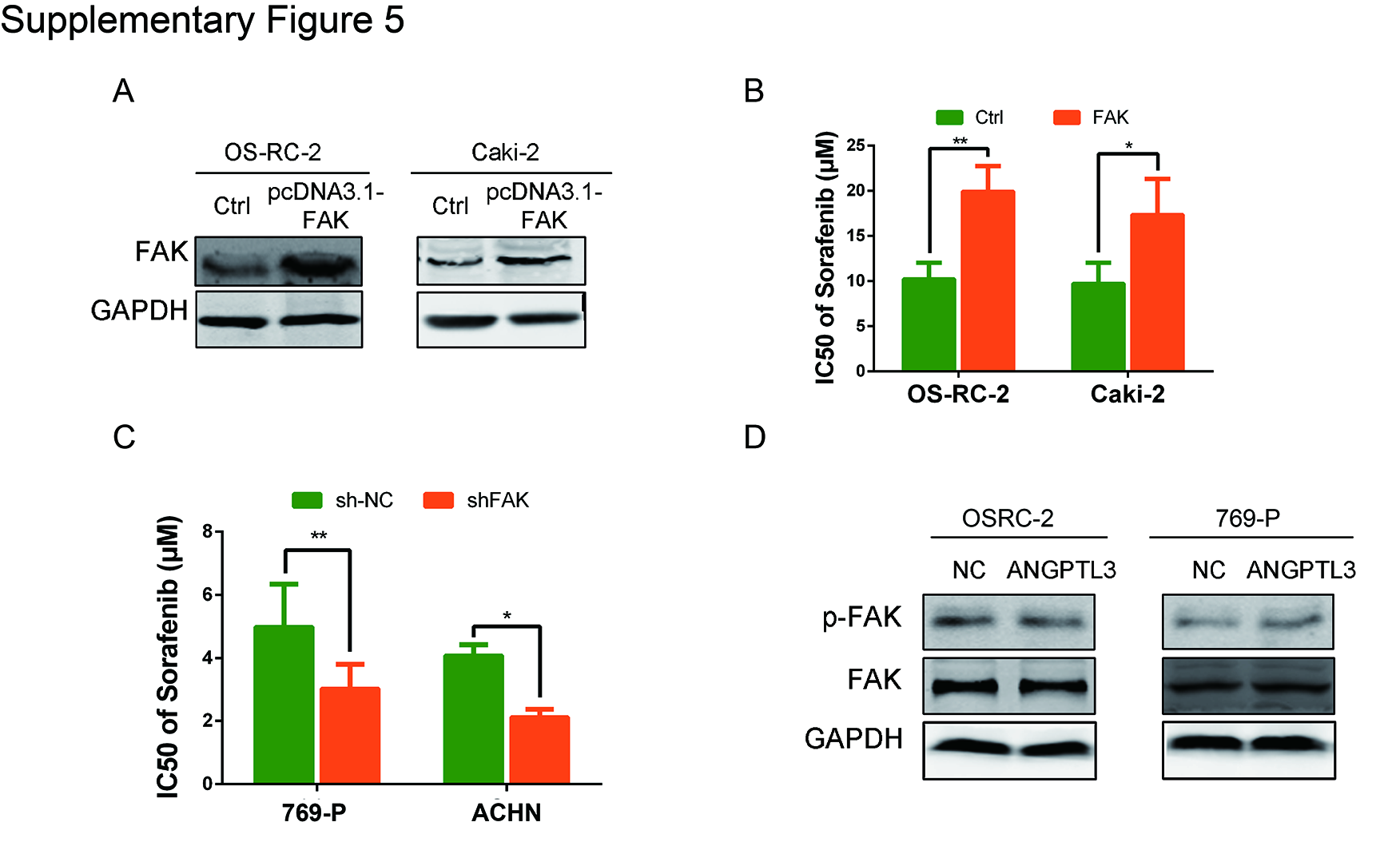

Supplement: Supplementary file 5 — Supplementary Figure 5 [file 41416_2018_189_MOESM5_ESM.tif]

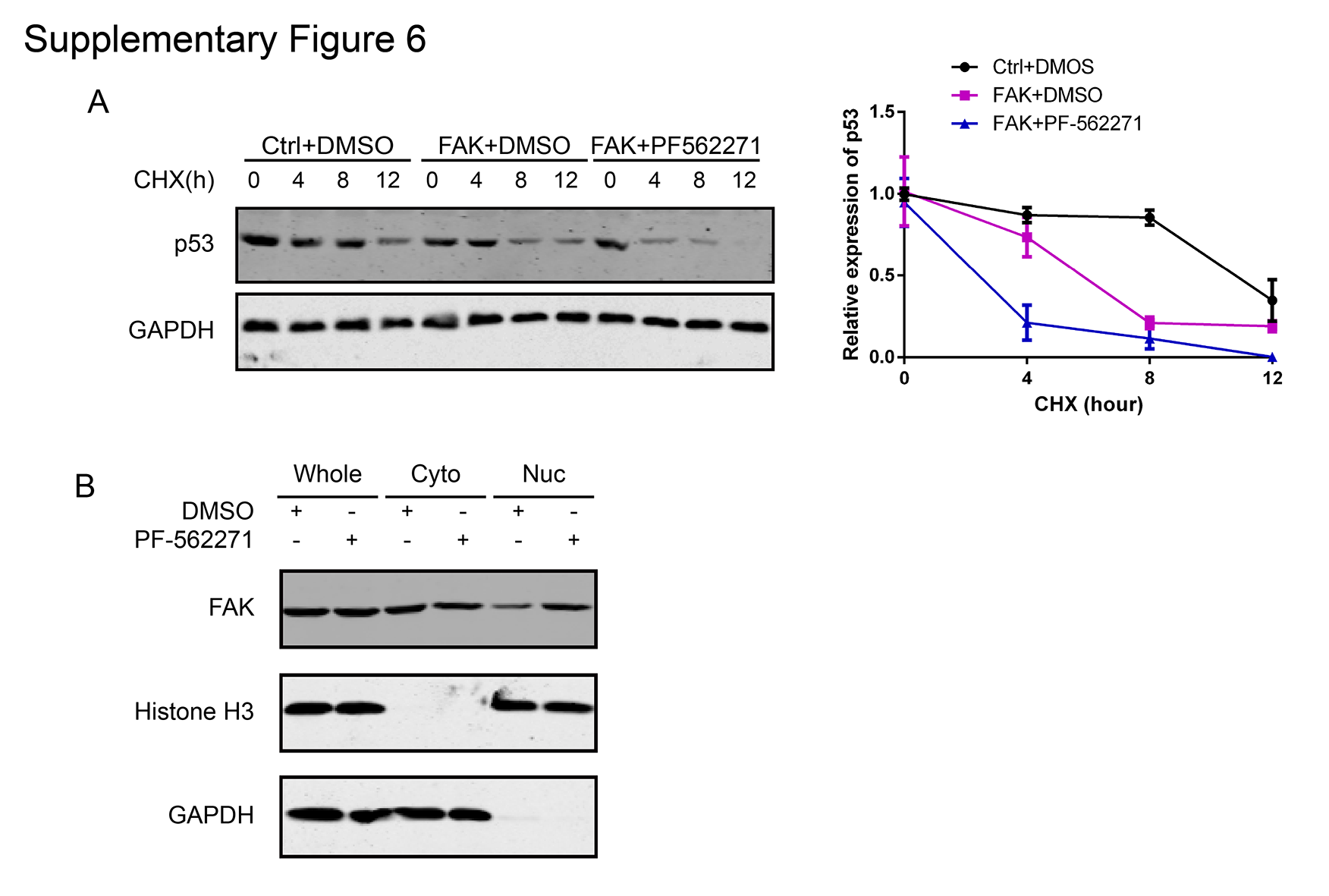

Supplement: Supplementary file 6 — Supplementary Figure 6 [file 41416_2018_189_MOESM6_ESM.tif]

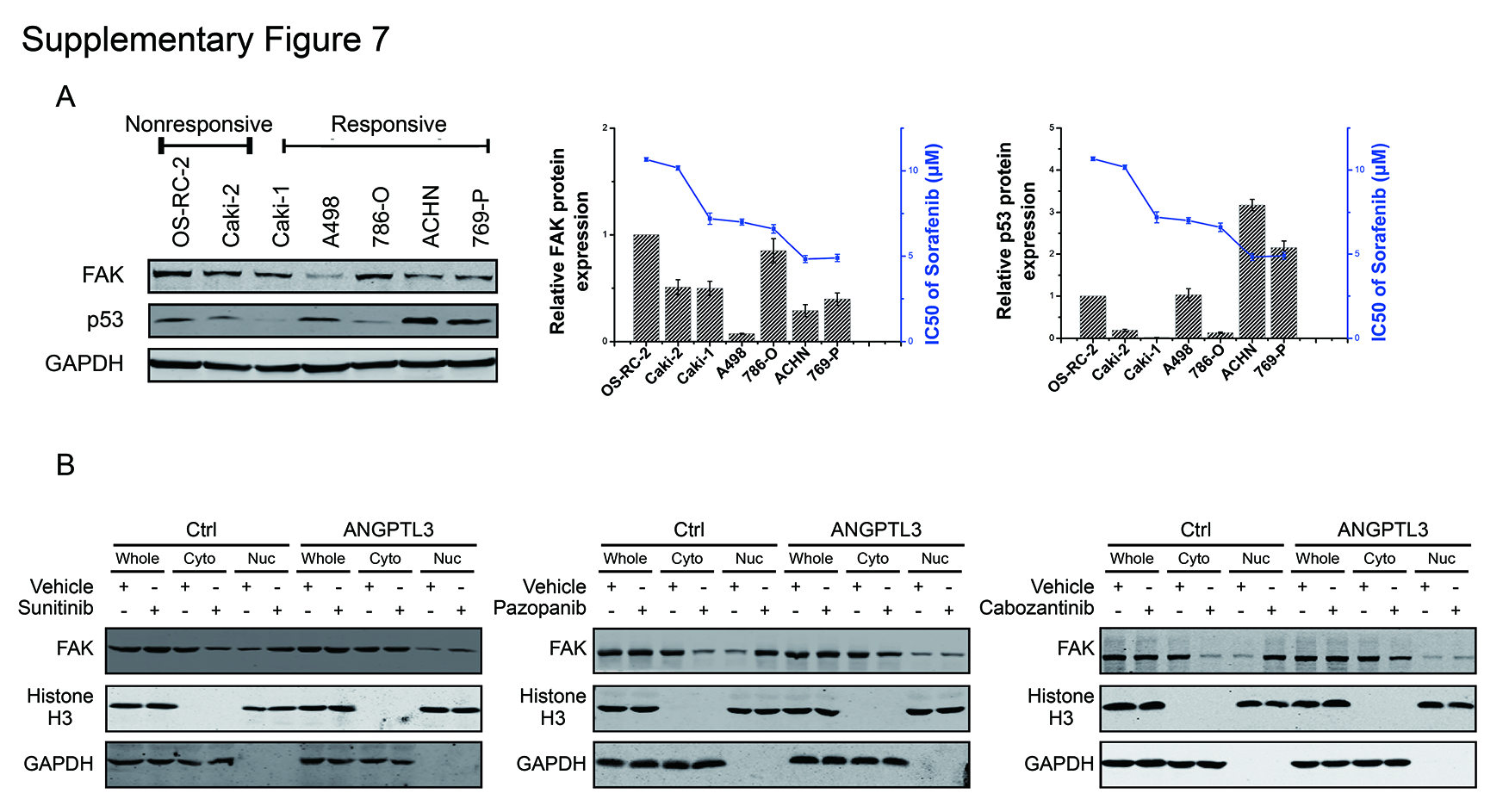

Supplement: Supplementary file 7 — Supplementary Figure 7 [file 41416_2018_189_MOESM7_ESM.tif]
