# Supplementary material for: Angiopoietin-like protein 3 blocks nuclear import of FAK and contributes to sorafenib response
Source: Br J Cancer. 2018 Jul 23;119(4):450–61. doi: 10.1038/s41416-018-0189-4 (PMC6134083; doi:10.1038/s41416-018-0189-4)
Supplement: Supplementary file 8 — Supplementary Figures legend clean vision [file 41416_2018_189_MOESM8_ESM.docx]

**Supplementary Figure 1, related to Figure 1. ANGPTL3 is preferentially upregulated in sorafenib-responsive renal cell carcinoma, and high ANGPTL3 levels predict better responses to sorafenib in RCC patients.** (A) The mRNA levels of ANGPTL 1, 2, 4, 5, 6, 7, 8 and the IC50 of sorafenib in 7 RCC cell lines (n=3). (B) The mRNA expression of ANGPTL 1, 2, 4, 5, 6, 7, 8 in an independent set of RCC tumors samples with good or poor responses to sorafenib therapy (n =3).

**Supplementary Figure 2, related to Figure 2. ANGPTL3 is required to maintain sorafenib sensitivity in RCC cells.** (A) qRT–PCR analysis of ANGPTL3 mRNA levels in ANGPTL3-knockdown and control 769p (left) and ACHN (right) cells (n=3). The IC50 values are shown in the lower histogram. (B) Western blot analysis of ANGPTL3 in ACHN and 769-P cells transfected with sh-ANGPTL3-1, sh-ANGPTL3-2 or control sh-NC after sorafenib treatment at the indicated concentrations for 48 h (n=3). GAPDH was used as a loading control.

Results are presented as the means ± SD. *p<0.05, **p < 0.01.

**Supplementary Fiugre 3, related to Figure 3. Overexpression of ANGPTL3 enhances the sorafenib sensitivity of RCC in vivo and in vitro.** (A) qRT-PCR analysis of ANGPTL3 mRNA levels in ANGPTL3-overexpressing and control OS-RC-2 (left) and caki-2 (right) cells (n=3). (B) Western blot analysis of ANGPTL3 in ANGPTL3-overexpressing and control OS-RC-2 and Caki-2 cells after sorafenib treatment at the indicated concentrations for 48 h (n=3). GAPDH was used as a loading control. (C) Representative immunohistochemical images of ANGPTL3-overexpressing and control OS-RC-2 xenografts in nude mice. The black scale bar represents 50 μm and the blue scale bar 500 μm. (D) The concentration of ANGPTL3 in the serum of nude mice. The serum of the tumour-bearing group was taken from nude mice given the vehicle before they were sacrificed, and the serum of the non-tumour-bearing group was taken from untreated nude mice of the same age.

Results are presented as the means ± SD. ***p<0.001.

**Supplementary Figure 4, related to Figure 4. ANGPTL3 physically interacts with FAK.** (A) ELISA analysis of ANGPTL3 in the cell culture supernatants and cell lysates of OS-RC-2 and 769-p cells. (B) Immunofluorescence analysis of ANGPTL3 (green) shows its subcellular distribution in 769-P cells. Scale bar, 50 μm. (C) Western blot analysis of ANGPTL3 in subcellular fractions of 769-p cells. (D) Verification of interference efficiency of siRNA for the indicated mRNA in 769-p and OS-RC-2 cells. (E) CCK8 assay of OS-RC-2 and 769-p cells transfected with the indicated siRNAs after sorafenib treatment at the indicated concentrations for 48 h (n=3). The IC50 values were calculated and are shown. (F) Western blot analysis of the indicated proteins in co-immunoprecipitation of ANGPTL3 in lysates of ANGPTL3-overexpressing OS-RC-2 cells.

Results are presented as the means ± SD. *p<0.05, **p<0.01

**Supplementary Figure 5, related to Figure 5. ANGPTL3 represses FAK-mediated sorafenib resistance and inhibits sorafenib-mediated FAK localization.** (A) Western blot analysis of the indicated proteins in FAK-overexpression and control RCC cells. (B) The IC50 of sorafenib for OS-RC-2 and Caki-2 cells transfected with pcDNA3.1-FAK or control pcDNA3.1. (C) The IC50 of sorafenib for 769-p and ACHN cells transfected with shFAK or sh-NC. (D) Western blot analysis of FAK and p-FAK proteins in ANGPTL3-overexpressing and control OS-RC-2 and Caki-2 cells.

Results are presented as the means ± SD. *p<0.05, **p<0.01

**Supplementary Figure 6, related to Figure 6. ANGPTL3 represses sorafenib resistance via inhibiting p53 ubiquitination.**

(A) Western blot analysis of p53 in FAK-overexpressing and control OS-RC-2 cells after cycloheximide (CHX) treatment together with PF-562271 (5 μM) or DMSO treatment for various times. (B) Western blot analysis of FAK in the whole lysate and subcellular fractions of OS-RC-2 cells after PF-562271 (5 μM) or DMSO treatment.

**Supplementary Figure 7, related to Figure 7. High ANGPTL3 levels predict better responses to sorafenib in RCC patients**

(A) Western blot analysis of FAK and p53 protein levels in the 7 RCC cell lines.The graph on the right shows FAK and p53 protein levels as well as the IC50 of sorafenib (n = 3). (B) Western blot analysis of FAK in the whole lysate and subcellular fractions of OS-RC-2 cells after sunitinib (2 μM), pazopanib (8μM), cabozantinib (5 μM) or vehicle treatment.
