# Supplementary material for: Angiopoietin-like protein 3 blocks nuclear import of FAK and contributes to sorafenib response
Source: Br J Cancer. 2018 Jul 23;119(4):450–61. doi: 10.1038/s41416-018-0189-4 (PMC6134083; doi:10.1038/s41416-018-0189-4)
Supplement: Supplementary file 19 — Supplementary Materials and Methods [file 41416_2018_189_MOESM19_ESM.docx]

**Supplementary Materials and Methods**

**1. RCC patients and clinical samples**

All patients provided written informed consent, and ethical consent was granted from the Committees for Ethical Review of Research involving Human Subjects of Second Military Medical University (Shanghai, China). The patient inclusion criteria for the sorafenib-treated and non-sorafenib-treated paired RCC tissues comprised a diagnosis of ccRCC and no prior anti-VEGF therapy. Patients who received prior immunotherapy (that is, received their targeted therapy as a second-line treatment) were also included. Patients treated initially with everolimus (an inhibitor of mammalian target of rapamycin, or mTOR), an investigational agent (that is, PTK787, AZD2171, or pazopanib), or an investigational combination (for example, bevacizumab plus erlotinib or bevacizumab plus sunitinib) were excluded. The presence of nodal and metastatic disease was defined according to intraoperative, pathologic and radiographic findings. Patients were staged using radiographic reports and postoperative pathological data and were reassigned according to the 2010 AJCC TNM classification.

**2.Real-time polymerase chain reaction (RT-PCR)**

Total RNA was isolated using TRIzol reagent (Invitrogen, Karlsruhe, Germany). First-strand cDNA was generated using the M-MLV Reverse Transcriptase (Invitrogen, Karlsruhe, Germany) and gene specific primers or random primers. Real-time PCR was performed in the StepOne™ Real-Time PCR System (Applied Biosystems, Foster City, USA) using SYBR® Green (Takara, Dalian, China) with gene specific primers (Supplementary Table S8). GAPDH mRNA was employed as an endogenous control for mRNA. The relative expression of RNAs was calculated using the comparative CT method.

**3.Western blot**

Cell lysates or retrieved proteins were analysed by immunoblot with primary antibodies and the secondary antibodies applied IRdye800-conjugated goat anti-rabbit IgG (Li-Cor Biosciences Inc., Lincoln, NE) and IRdye700-conjugated goat anti-mouse or anti-rabbit IgG (Li-Cor Biosciences Inc., Lincoln, NE) and were detected using an Odyssey infrared scanner (Li-Cor Biosciences Inc., Lincoln, NE). Primary antibodies used in this study are listed in Supplementary Table S9.

**4.ANGPTL3 ELISA**

ANGPTL3 levels in the cell lysis and in the normal culture medium collected for 48 hours from different cells were detected with the Human ANGPTL3 ELISA Kit (AdiopGen, the United States) according to the manufacturer’s instructions.

**5.Cell Counting Kit‑8 (CCK‑8) assay**

RCC cells were seeded at a density of 6 × 103 cells per well in 96-well plates, and the viability of the cells was assessed from 3 replicates in 3 independent experiments by the Cell Counting kit-8 (Dojindo Laboratories, Japan) after treated with indicated reagents at indicated concentration for 48 hours.

**6.Apoptotic analysis with Annexin V Staining**

Cells were seeded in a 6-well plate at a density of 2.0 x 105 cells/well and treated with indicated reagents at indicated concentration for 48 hours. Then cells were collected, washed twice with ice-cold PBS ,and 1.0 x 106 cells were resuspended in 1 ml 1 X binding buffer, 100μL cell suspension was transferred to a 5 ml culture tube and labeled with 5 μl fluoresceinisothiocyanate(FITC)-conjugated annexin V and 5 μl propidium iodine according to the manufacturer's instructions (556547, BD Pharmingen™, United States). After incubated at RT(25℃) in dark for 15min, the samples were supplemented with 400μL 1× binding buffer, then were immediately analyzed on a flow cytometer (MACSQuant, Miltenyi Biotec, Germany)within 1h.
